# Multimodality MRI assessment of grey and white matter injury and blood-brain barrier disruption after intracerebral haemorrhage in mice

**DOI:** 10.1038/srep40358

**Published:** 2017-01-13

**Authors:** Jie Yang, Qian Li, Zhongyu Wang, Cunfang Qi, Xiaoning Han, Xi Lan, Jieru Wan, Wenzhu Wang, Xiaochun Zhao, Zhipeng Hou, Cong Gao, J. Ricardo Carhuapoma, Susumu Mori, Jiangyang Zhang, Jian Wang

**Affiliations:** 1 Key Laboratory of Neurogenetics and Channelopathies of Guangdong Province and The Ministry of Education of China, Institute of Neuroscience, Department of Neurology, the Second Affiliated Hospital of Guangzhou Medical University, Guangzhou, China; 2 Department of Anesthesiology/Critical Care Medicine, The Johns Hopkins University, School of Medicine, Baltimore, MD 21205, USA; 3 Department of Radiology, The Johns Hopkins University School of Medicine, Baltimore, MD 21205, USA; 4 Department of Neurology, The Johns Hopkins University School of Medicine, Baltimore, MD 21205, USA; 5 Bernard and Irene Schwartz Center for Biomedical Imaging, Department of Radiology, New York University School of Medicine, New York, NY 10016, USA

## Abstract

In this study, we examined injury progression after intracerebral haemorrhage (ICH) induced by collagenase in mice using a preclinical 11.7 Tesla MRI system. On T2-weighted MRI, lesion and striatal volumes were increased on day 3 and then decreased from days 7 to 28. On day 3, with an increase in striatal water content, vasogenic oedema in the perihaematomal region presented as increased T2 and increased apparent diffusion coefficient (ADC) signal. With a synchronous change in T2 and ADC signals, microglial activation peaked on day 3 in the same region and decreased over time. Iron deposition appeared on day 3 around the haematoma border but did not change synchronously with ADC signals. Vascular permeability measured by Evans blue extravasation on days 1, 3, and 7 correlated with the T1-gadolinium results, both of which peaked on day 3. On diffusion tensor imaging, white matter injury was prominent in the corpus callosum and internal capsule on day 3 and then partially recovered over time. Our results indicate that the evolution of grey/white matter injury and blood-brain barrier disruption after ICH can be assessed with multimodal MRI, and that perihaematomal vasogenic oedema might be attributable to microglial activation, iron deposition, and blood-brain barrier breakdown.

Spontaneous intracerebral haemorrhage (ICH) remains a significant cause of morbidity and mortality throughout the world^1^. Its mortality increases from 40% at 1 month to 54% at 1 year^2^. However, interventions for ICH are very limited. Therefore, understanding the mechanisms of ICH pathology and repair and identifying potential treatments have high priorities.

Advances in imaging options, especially in magnetic resonance imaging (MRI), have resulted in improved management and prognostication of ICH^3^. T2-weighted (T2wt) MRI is used to define the area of blood clot in the haemorrhagic lesion^4^; T1-gadolium (T1-Gd) enhancement MRI can detect the presence of blood-brain barrier (BBB) disruption^5^; apparent diffusion coefficient (ADC) can be used to evaluate cytotoxic and vasogenic oedema^6^,^7^; and diffusion tensor imaging (DTI) allows for quantitative evaluation of the structural integrity of white matter tracts, which can predict motor recovery^8^,^9^.

Preclinical ICH models have greatly improved our knowledge of the pathophysiology of ICH and have been a valuable tool for testing potential therapeutic strategies. In particular, transgenic mouse models are helpful for addressing genetic influences on early brain injury and late brain repair after ICH^10^. As one of the two most commonly used ICH models, collagenase injection is a simple and reproducible procedure that lacks the drawbacks associated with the blood injection model such as variable lesion size caused by backflow of the injected blood along the needle track^10^,^11^,^12^. Furthermore, in contrast to the blood ICH model, the collagenase model mimics an acute cerebrovascular rupture and BBB breakdown and imitates haematoma expansion^13^ similar to that present in ICH patients^10^,^12^. The blood ICH model has advantages for investigating the mechanisms of blood clot toxicity.

Because elucidating pathogenic mechanisms and testing potential therapies are the focus of current preclinical ICH research, it is important to use clinically relevant MRI parameters to assess the evolution of ICH-induced brain damage and repair in the same subject. In recent studies, investigators have reported MRI-assessed lesion volume and brain tissue loss by T2wt MRI^14^,^15^,^16^, brain oedema volume by ADC^17^, white matter injury by DTI^18^,^19^, and BBB disruption by T1-Gd MRI^20^, mostly in rats. None of the studies has used multimodality MRI to assess the grey and white matter injury and repair, perihaematomal oedema, or BBB disruption over time in a mouse ICH model. In this study, we used T2wt MRI, T1-Gd MRI, and DTI to investigate corresponding changes in brain structure on days 3, 7, and 28 in a collagenase-induced mouse model of ICH. We also compared these MRI features to histologic changes and investigated the correlation between MRI findings and neurologic deficits.

## Results

### Changes in haematomal lesion volume on T2wt MRI and histology

**Figure 1:**
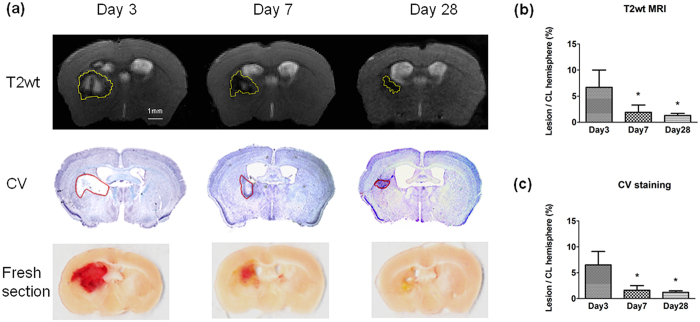
Haemorrhagic lesion resolution assessed by T2wt MRI and cresyl violet (CV) staining. (**a**) Representative images show haemorrhagic lesion on T2wt MRI (top, yellow outline), CV-stained brain slices (middle, red outline), and adjacent fresh brain sections (unstained, bottom) at 3, 7, and 28 days after ICH. Different cohorts of animals were used for MRI scans and for histology. (**b**,**c**) Quantitative analysis shows that the time-dependent changes in ratio of total lesion volume to the contralateral hemisphere volume were similar when assessed by T2wt-MRI and CV. MRI: n = 8 at each time point; CV: n = 6 at each time point; fresh section: one at each time point (*P < 0.05 vs. day 3; one-way ANOVA). CL = contralateral.

Haematomal lesion volume was manually delineated and measured in T2wt MRI and cresyl violet (CV)-stained brain sections. At day 3, T2wt MRI showed that the lesions contained a region with high signal intensities surrounded by dark rims. The signal in the high-intensity region was significantly reduced on days 7 and 28 (Fig. 1a). On the CV-stained brain sections, lesions or dead tissues were identified by lack of staining on days 3 and 7, and a fibrous scar was identified on day 28 (Fig. 1a). Adjacent fresh brain sections showed that the haematoma in the striatum resolved over the course of 28 days (Fig. 1a). Both T2wt MRI and CV staining showed a progressive decrease in relative lesion volume (percent of contralateral hemisphere; day 3: 6.7 ± 3.3% and 6.5 ± 2.6%; day 7: 1.9 ± 1.4% and 1.6 ± 0.9%; day 28: 1.3 ± 0.4% and 1.2 ± 0.4%; Fig. 1b,c).

### Volumetric changes after ICH

**Figure 2:**
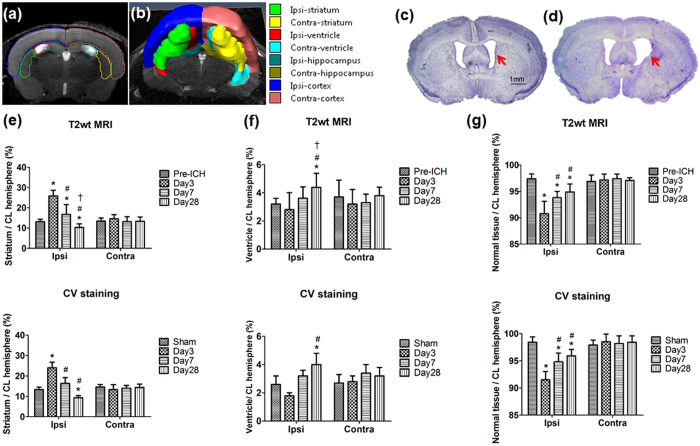
Brain region volume changes over time after ICH. Volumetric measurements of different brain regions were made on T2wt MRI (**a**,**b**) and on cresyl violet (CV)-stained brain sections for comparison (**c**,**d**). Volumetric measurement showed ventricle extension at day 28 (**d**) compared with pre-ICH normal ventricle (**c**; red arrows). (**e**) Quantitative analysis of both T2wt MRI (top) and CV staining (bottom) showed that the ratio of ipsilateral striatum to contralateral hemisphere volume increased significantly at day 3 and then progressively decreased at days 7 and 28. (**f**) The ratio of ipsilateral ventricle to contralateral hemisphere decreased at day 3 and then progressively increased at days 7 and 28 on both T2wt MRI and CV staining. (**g**) The ratio of normal tissue in the ipsilateral hemisphere to contralateral hemisphere decreased at day 3 and then progressively increased at days 7 and 28 on both MRI and CV staining, showing that tissue loss persisted from the acute stage to the chronic stage (T2wt MRI: n = 5 for pre-ICH, n = 8 for other time points; CV staining: n = 6 at each time point; *P < 0.05 vs. pre-ICH; ^#^*P* < 0.05 vs. day 3; ^†^*P* < 0.05 vs. day 7, one-way ANOVA). CL = contralateral.

Based on T2wt MRI, we obtained the volumes of major structures in the ICH brain via segmentation (Fig. 2a,b). The relative volume of the ipsilateral striatum (percent of contralateral hemisphere) increased significantly from 13.1 ± 1.2% pre-ICH to 25.9 ± 2.7% at 3 days post-ICH and then decreased to 16.8 ± 4.8% at 7 days and 10.3 ± 1.8% at 28 days (all *P* < 0.001 vs. pre-ICH; Fig. 2e, top panel). These changes paralleled the changes in CV staining at the corresponding time points (pre-ICH, 13.4 ± 1.2%; day 3, 24 ± 2.8%; day 7, 16.4 ± 2.8%; and day 28, 9.4 ± 1%; all *P* < 0.05 vs. pre-ICH; Fig. 2e, bottom panel). The ipsilateral ventricular volume decreased from 3.20 ± 0.4% pre-ICH to 2.81 ± 1.2% at 3 days and then increased to 3.62 ± 0.8% at 7 days and 4.39 ± 1% at 28 days (*P* < 0.05; Fig. 2c,d; Fig. 2f, top), again paralleled by changes in CV staining (2.6 ± 1.2%, 1.8 ± 0.2%, 3.2 ± 0.8%, and 4.0 ± 0.8%) at the respective time points (Fig. 2f, bottom). The *post hoc* test showed that ventricular volume at day 28 was significantly larger than that pre-ICH (*P* = 0.011), whereas striatal volume was significantly reduced at that time point (*P* = 0.04). The tissue loss in the ipsilateral parenchyma showed significant reduction at day 3 (90.8 ± 2.3%), day 7 (93.8 ± 1.2%), and day 28 (94.9 ± 1.5%) compared to pre-ICH (97.4 ± 0.9%; *P* < 0.001; Fig. 2g, top) and was paralleled by changes in CV staining at the corresponding time points (91.5 ± 1.5%, 94.8 ± 1.6%, 95.9 ± 1.2%, and 98.4 ± 1%; Fig. 2g, bottom). We also found significantly decreased volume in the ipsilateral cortex at day 28 compared with that pre-ICH on T2wt MRI but no changes in CV staining. There was no change in hippocampal volumes by either MRI or CV staining (See Supplementary Fig. S1).

### Perihaematomal oedema

**Figure 3:**
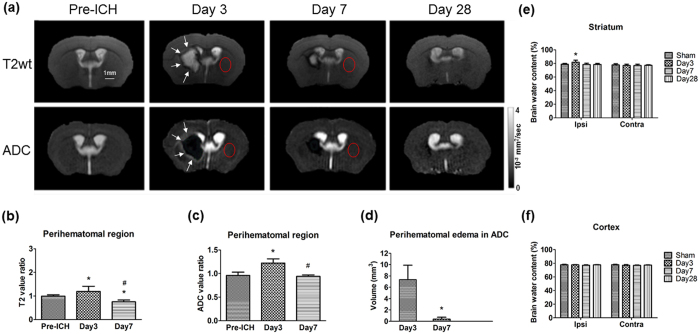
*In vivo* changes in brain oedema shown by T2wt MRI and apparent diffusion coefficient (ADC). (**a**) On days 3 and 7 after ICH, T2wt MRI showed a hyperintense region in the lesion core, whereas ADC images showed hypointense signal in the corresponding brain region, suggestive of dead tissue. In the perihaematomal region, imaging showed an isointense to hyperintense edge on T2wt MRI and elevated ADC values in the corresponding brain region (arrows), suggestive of vasogenic oedema. The red outline on T2wt MRI and ADC images shows the region of interest on the contralateral hemisphere that was chosen for quantitative analysis. (**b**) The ratio of T2 value in the perihaematomal region to that in the contralateral normal tissue was elevated on day 3, but decreased on day 7, compared with that at baseline. (**c**) The ratio of ADC value in the perihaematomal region was elevated on day 3, but not on day 7, compared with that at pre-ICH, indicating that perihaematomal vasogenic oedema increased initially but was gone by day 7. (**d**) The volume of perihaematomal oedema in ADC decreased significantly from day 3 to day 7. (**b,c:** n = 5 for pre-ICH, n = 8 for other time points; *P < 0.05 vs. pre-ICH; ^#^*P* < 0.05 vs. day 3 post-ICH, one-way ANOVA; **d**: n = 8 for each group, Student t-test). (**e**) Measurement of brain water content in ipsilateral striatum showed a peak at day 3 and then a decrease to baseline at day 7. Contralateral striatum showed no change in water content. (**f**) Brain water content remained unchanged in cortex bilaterally. (**e,f:** n = 4 for sham and day 28 post-ICH; n = 7 for days 3 and 7 post-ICH; *P < 0.05 vs. pre-ICH; ^#^*P* < 0.05 vs. day 3 post-ICH, one-way ANOVA).

Tissue ADC estimated from diffusion MRI measurements has traditionally been used to detect cytotoxic and vasogenic oedema after stroke. Cytotoxic oedema represents the redistribution of water from extracellular to intracellular compartments, without a change in local constituents; therefore, MRI T1 and T2 signal intensity should not change markedly. Because cytotoxic oedema with minimum changes in T2 signal and large decreases in ADC signal cannot be detected on days 3 and 7 in our model, we quantified the volume of vasogenic oedema in the perihaematomal region with high T2 and high ADC signal^21^ (Fig. 3a, arrows). We used the ratio of values in the perihaematomal region to values in the corresponding contralateral brain region (Fig. 3a, red circle) on T2 or ADC images to show how the signal changed from the pre-ICH time point to days 3 and 7 post-ICH. The ratio of T2 values in the perihaematomal region was increased significantly at day 3 and then decreased at day 7 compared to that at baseline (0.99 ± 0.06, 1.19 ± 0.22, and 0.75 ± 0.08 at baseline, day 3, and day 7, respectively, *P* < 0.001 on day 3; Fig. 3b). The ratio of ADC values in the perihaematomal region was significantly higher at day 3 but decreased to the baseline level at day 7, compared to that at pre-ICH (0.96 ± 0.07, 1.22 ± 0.09, and 0.94 ± 0.03 at baseline, day 3, and day 7, respectively, *P* < 0.001 on day 3; Fig. 3c). Measurements of brain oedema showed that brain water content in the ipsilateral striatum increased significantly from 78.7 ± 1.4% at pre-ICH to 81.7 ± 3.1% on day 3 (*P* = 0.026), and returned to baseline at days 7 (78.4 ± 2.0%) and 28 (78.5 ± 1.6%). The volume of perihaematomal regions with hyperintense ADC signals indicative of vasogenic oedema decreased from day 3 (7.36 ± 2.55 mm^3^) to day 7 (0.37 ± 0.34 mm^3^, *P* < 0.001; Fig. 3d). In comparison, the contralateral striatum, ipsilateral cortex, and contralateral cortex showed no significant change in tissue water content during the same time period (Fig. 3e,f), suggesting that brain oedema increases transiently in the ipsilateral striatum at day 3.

### T2 and ADC signals change in both lesion and perilesional areas

In addition to clearly defined brain regions with perihaematomal vasogenic oedema, the unique signatures in MRI signals, combined with T2 and ADC signals, identified another four brain regions associated with the lesion that may experience different pathologic states. These regions include lesion core, lesion edge, perihaematomal region, and oedema edge (see Supplementary Fig. S2a). We used the ratio of values in these four areas to that in the contralateral brain tissue on T2 or ADC images to show the change in signals before ICH and at days 3 and 7. In the lesion core, the ratio of T2 value was elevated at day 3 and decreased at day 7, but at both time points, it was higher than the pre-ICH value (0.99 ± 0.06, 2.12 ± 0.62, and 1.57 ± 0.34 at baseline, day 3, and day 7, respectively, all *P* < 0.05). The ratio of ADC value decreased significantly from day 3 to day 7 compared to that at pre-ICH (0.96 ± 0.06, 0.66 ± 0.02, and 0.52 ± 0.1 at baseline, day 3, and day 7, respectively, all *P* < 0.05) (see Supplementary Fig. S2b). At lesion edge, the ratio of both T2 and ADC value decreased significantly from day 3 to day 7 compared with that at pre-ICH (T2: 0.99 ± 0.06, 0.78 ± 0.1, and 0.42 ± 0.18 at baseline, day 3, and day 7, respectively, all *P* < 0.05; ADC: 0.96 ± 0.07, 0.73 ± 0.21, and 0.53 ± 0.09, respectively, all *P* < 0.05; Supplementary Fig. S2c). The quantification of the signal change in the perihaematomal region (vasogenic oedema with high T2 and high ADC signals, Supplementary Fig. S2d) is shown in Fig. 3a–d. At oedema edge, the ratio of T2 value did not change at day 3 but decreased at day 7 compared to that at pre-ICH (0.99 ± 0.06, 0.94 ± 0.08, and 0.83 ± 0.05 at baseline, day 3, and day 7, respectively, *P* = 0.001). The ratio of ADC value increased significantly at day 3 but decreased at day 7 compared to that at pre-ICH (0.96 ± 0.07, 1.05 ± 0.06, and 0.97 ± 0.02 at baseline, day 3, and day 7, respectively, *P* = 0.01; see Supplementary Fig. S2e).

### Perihaematomal inflammation

**Figure 4:**
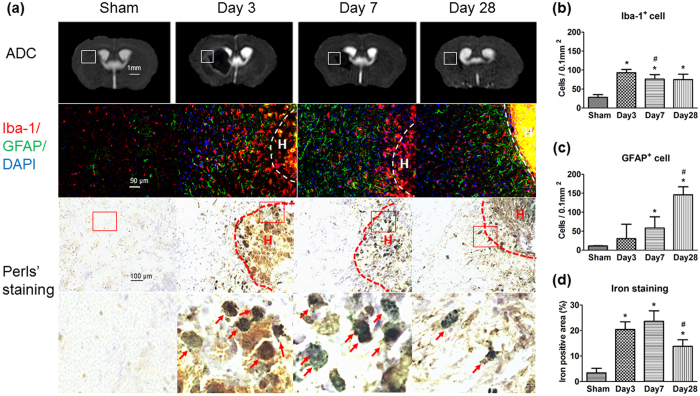
Glial response and iron deposition in the perihaematomal region. Iba-1/GFAP immunostaining was used to identify glial response, and Perls’ staining was used to identify iron deposition along the inner border of the haematoma. (**a**) Areas of staining were compared to the perihaematomal hyperintensive regions on ADC (top row). High magnification images of the Perls’ staining in the bottom row were from the boxed areas in the third row. H, haematoma; (**b**) Quantitative analysis showed that activated Iba-1-positive cells significantly increased after ICH, especially at the border zone of the haemorrhagic lesion from day 3 to day 28, as compared to that in the sham group. The number of activated Iba-1-positive cells was lower on days 7 and 28 than on day 3. (**c**) The number of GFAP-positive cells increased progressively from day 3 to day 28. (**d**) Perls’ staining showed large amounts of iron-positive cells (red arrows in A) along the inner border of the haematoma at days 3 and 7, most of which were activated microglia/macrophages based on cell morphology and cell body diameter. Quantitative analysis showed that the iron-positive area increased from day 3 to day 7 but decreased at day 28 after ICH. The staining results illustrate that microglial activation and iron deposition may contribute to the hyperintensive signal on ADC in the perihaematomal region. (ADC: n = 5 for pre-ICH, n = 8 at other time points; immunostaining: n = 5; *P < 0.05 vs. pre-ICH; ^#^*P* < 0.05 vs. day 3, one-way ANOVA).

Activated microglia positive for Iba-1 significantly increased after ICH (indicative of neuroinflammation), especially in the perihaematomal region on day 3. The area with increased Iba-1 signal intensity approximately corresponded to the regions with hyperintense ADC signals (Fig. 4a, first and second rows). The number of activated microglia increased around the lesion at 3 days and then decreased at 7 and 28 days (28 ± 7/0.1 mm^2^, 93 ± 9/0.1 mm^2^, 76 ± 12/0.1 mm^2^, and 75 ± 14/0.1 mm^2^ for sham and at days 3, 7, and 28 post-ICH, respectively, all *P* < 0.05 vs. sham, Fig. 4a second row and b). The number of GFAP-positive cells progressively increased from day 3 to day 28, compared with that in sham animals (11 ± 0.8/0.1 mm^2^, 30 ± 38/0.1 mm^2^, 58 ± 30/0.1 mm^2^, and 146 ± 21/0.1 mm^2^ for sham and days 3, 7, and 28 post-ICH, respectively, all *P* < 0.05, Fig. 4a second row and c). Perls’ staining (indicative of iron deposition) showed large numbers of iron-positive cells along the inner border of the haematoma at days 3 and 7; most appeared to be activated microglia/macrophages, based on cell morphology (Fig. 4a third and fourth row). Quantification analysis showed that the iron-positive area increased significantly after ICH compared with that of the sham group (3.4 ± 1.8%, 20.4 ± 3.0%, 23.6 ± 4.1%, and 13.8 ± 2.6% for sham and days 3, 7, and 28 post-ICH, respectively, *P* = 0.02, Fig. 4d). These results indicate that glial response, especially microglia/macrophage activation and iron deposition, may play a role in the formation of perihaematomal oedema.

### BBB disruption

**Figure 5:**
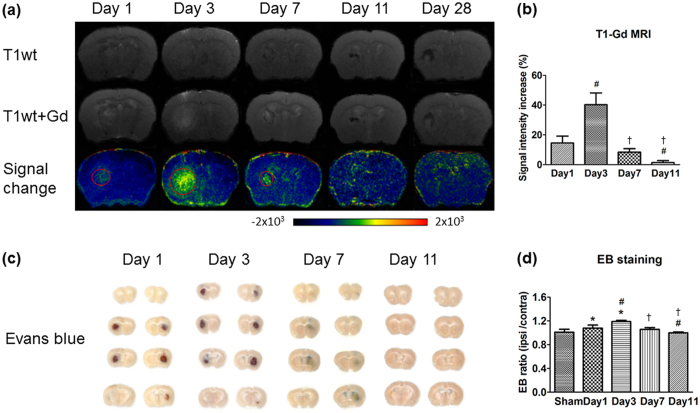
BBB breakdown as shown by enhanced T1wt and Evans blue leakage. (**a**) Representative T1wt MRI before (top) and after (middle) gadolinium (Gd) administration on days 1, 3, 7, 11, and 28 post-ICH. Bottom: the intensity of signal change increased from day 1 to day 3 and then decreased progressively from day 3 to day 11. Almost no enhancement remained on days 11 and 28. (**b**) Quantitative analysis showed that Gd enhancement declined progressively from day 3 to day 11, indicating that BBB recovery began by day 7 and was nearly complete at day 11 (n = 5 for each time point; ^#^*P* < 0.05 vs. day 1; ^†^*P* < 0.05 vs. day 3, one-way ANOVA). (**c**) Evans blue staining showed that BBB leakage was present from day 1 to day 7 after ICH but had nearly stopped at day 11. (**d**) Quantitative analysis showed that Evans blue (EB) concentration peaked at day 3, declined from day 7, and reached the level of sham-operated mice at day 11. The trend was similar to that observed with T1-Gd MRI (n = 4 for each time point; *P < 0.05 vs. sham, ^#^*P* < 0.05 vs. day 1, ^†^*P* < 0.05 vs. day 3, one-way ANOVA).

We estimated BBB breakdown by both gadolinium-enhanced T1-MRI (T1-Gd MRI) and Evans blue staining. In T1-Gd MRI, the signal intensity increased from day 1 (14.5 ± 4.7 with respect to signals from the contralateral side) to day 3 (40.3 ± 7.9), and then decreased progressively from day 3 to days 7 and 11 (8.4 ± 2.3 and 1.4 ± 1.3, respectively, both *P* < 0.05 vs. day 3; Fig. 5a,b). No apparent enhancement was visible on days 11 and 28, indicating that BBB recovery started from day 7 and was nearly complete by day 11. The MRI findings were confirmed by Evans blue staining, which showed an increase in leakage on days 1 to 7 after ICH that had nearly stopped by day 11. Quantitative analysis showed that the ratio of Evans blue concentration (ipsilateral/contralateral hemisphere) peaked at day 3 (1.19 ± 0.02), began to decline at day 7 (1.06 ± 0.03), and reached the level of sham controls (1.04 ± 0.11) on day 11 (1.0 ± 0.01, *P* < 0.05 vs. day 3), similar to the trend seen on T1-Gd MRI (Fig. 5c,d).

### White matter injury

**Figure 6:**
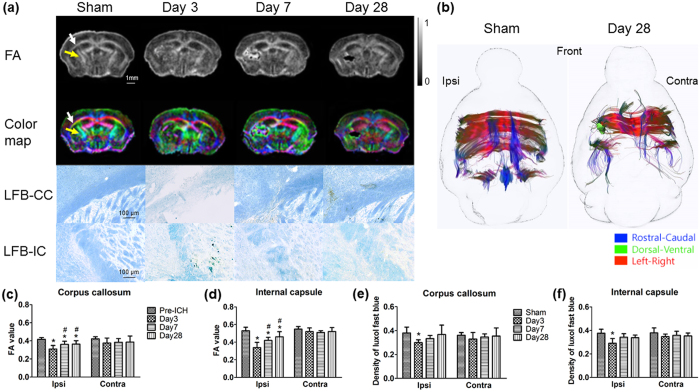
White matter injury measured by fractional anisotropy (FA) and Luxol fast blue (LFB) staining. (**a**) Representative FA images and colour maps show white matter injury of the ipsilateral corpus callosum (CC) and internal capsule (IC) on days 3, 7, and 28 after ICH. Images from sham-operated mice are shown for comparison. Arrows indicate the segments of CC (white arrows) and IC (yellow arrows) measured. LFB staining shows intact myelin of the ipsilateral CC and IC at corresponding time points. (**b**) In *ex vivo* DTI, the fibre tracts in CC were blocked or interrupted by lesion (green mass), and the ipsilateral fibre volume was significantly less than that in sham mice on day 28, indicating that white matter fibre loss is not fully recovered at that time point. (**c,d**) Quantification analysis shows that FA values of the ipsilateral CC (**c**) and IC (**d**) were significantly decreased on day 3 but then progressively improved on days 7 and 28. (**e,f**) Quantification analysis of LFB staining shows that the time course of myelin loss was the same as that seen with FA. (FA: n = 5 for pre-ICH, n = 8 at other time points; LFB: n = 6; *P < 0.05 vs. pre-ICH; ^#^*P* < 0.05 vs. day 3, one-way ANOVA).

Both fractional anisotropy (FA; Fig. 6a, top row) and *ex vivo* DTI (Fig. 6b) showed white matter injury after ICH. At baseline, the FA value of the ipsilateral corpus callosum (CC) was 0.42 ± 0.02. After ICH, the FA values were significantly reduced at days 3, 7, and 28 (all *P* < 0.05; Fig. 6a,c). Changes in the ipsilateral internal capsule (IC) showed the same trend, with FA value decreasing from 0.55 ± 0.03 at baseline to 0.34 ± 0.06, 0.42 ± 0.03, and 0.46 ± 0.06 on days 3, 7, and 28, respectively (all *P* < 0.05; Fig. 6a,d). Luxol fast blue (LFB) staining provided similar results. The LFB density on brain sections in the ipsilateral CC was 0.38 ± 0.05 at baseline and then decreased to 0.30 ± 0.02, 0.33 ± 0.03, and 0.37 ± 0.08 on days 3, 7, and 28, respectively (all *P* < 0.05; Fig. 6e). In the ipsilateral IC, the LFB density was 0.38 ± 0.04 at baseline and then decreased to 0.29 ± 0.04, 0.34 ± 0.03, and 0.34 ± 0.02 at the corresponding time points (all *P* < 0.05; Fig. 6f). In both CC and IC, FA and LFB results showed significant decreases in the signal intensity at day 3 followed by a progressive repair at days 7 and 28. Neither FA nor LFB showed a significant change in the contralateral IC and CC (Fig. 6c–f). Tractography results based on the *ex vivo* DTI data showed disrupted white matter tracts on the ipsilateral side in a mouse brain at 28 days after ICH, which indicates that white matter injury was not recovered at that time point (Fig. 6b). Since cerebral peduncle (CP) is associated with clinical outcome in ICH patients, we quantified the FA value in CP in our model. Results from the ipsilateral hemisphere showed lower FA value in CP from day 3 to day 28 post-ICH compared with that in the sham group (0.68 ± 0.04, 0.59 ± 0.06, 0.60 ± 0.07, and 0.61 ± 0.06 for sham and days 3, 7, and 28 post-ICH, respectively; all *P* < 0.05; Supplementary Fig. S3a,b). We also analysed the intensity of LFB staining in the striatum around the lesion. The intensity of LFB staining on the ipsilateral side was significantly decreased on days 3 and 7 after ICH, but not on day 28, compared with that in the sham group (0.38 ± 0.04, 0.30 ± 0.02, 0.32 ± 0.03, and 0.36 ± 0.05 for sham and days 3, 7, and 28 post-ICH, respectively; *P* < 0.05 vs. sham at day 3 and day 7; *P* = 0.423 vs. sham at day 28; Supplementary Fig. S3c). Neither FA nor LFB showed a significant change in the contralateral CP or striatum (Supplementary Fig. S3b,c). Using transmission electron microscopy, we showed marked pathologic changes in myelin ultrastructure characterized by myelin breakdown or disruption at day 3 post-ICH compared with intact myelin in the sham mouse brain (Supplementary Fig. S3d).

### Correlation between neurologic deficit score (NDS) and MRI signal changes

**Figure 7:**
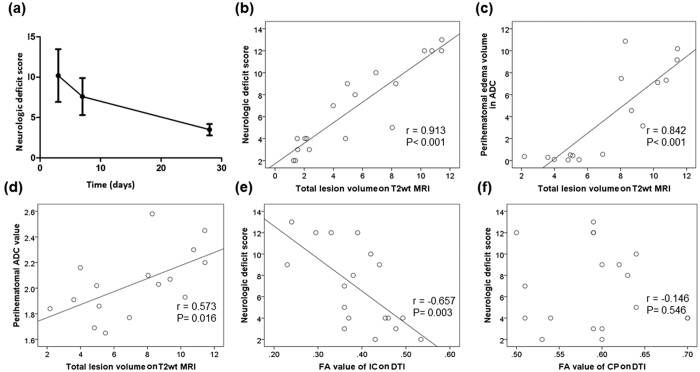
Neurologic deficit score (NDS) correlates with lesion volume and white matter injury assessed by MRI. (**a**) Neurologic deficit progressively decreased from day 3 to day 28 after ICH (n = 6 mice/group). (**b**) Scatter plot shows positive correlation between total lesion volume on T2 MRI and NDS. (**c**,**d**) Scatter plots show positive correlation between perihaematomal oedema volume (**c**) predicted by apparent diffusion coefficient (ADC) or perihaematomal ADC value (**d**) and total lesion volume on T2wt MRI. (**e**) Scatter plot shows negative correlation between fractional anisotropy (FA) value of ipsilateral internal capsule (IC) and NDS. This correlation disappeared after adjusting for total lesion volume on T2wt MRI. (**f**) No correlation was apparent between the FA of cerebral peduncle (CP) in DTI MRI and NDS. n = 6 for each time point.

The NDS decreased from day 3 (10.2 ± 3.3) to day 7 (7.6 ± 2.3) and day 28 (3.5 ± 0.7; all *P* < 0.001; Fig. 7a). Correlation between NDS and lesion volume was positive, with a Pearson correlation coefficient of 0.913 (*P* < 0.001; Fig. 7b). The correlation between perihaematomal oedema volume in ADC and total lesion volume in T2wt MRI was positive (*P* < 0.001; Fig. 7c), as was the correlation between perihaematomal ADC value and total lesion volume in T2wt MRI (*P* = 0.016, Fig. 7d). We found a negative correlation between NDS and FA value of the ipsilateral IC on DTI MRI (*P* = 0.003, Fig. 7e) that disappeared after we adjusted for total lesion volume on T2 MRI. We found no correlation between NDS and FA value of the ipsilateral CP on DTI MRI (*P* = 0.546, Fig. 7f).

## Discussion

In this study, we investigated whether noninvasive MRI could be used to evaluate the severity of grey and white matter injury and BBB disruption in a mouse model of ICH by cross-examining MRI and histologic results. Our study is novel in several ways: (1) We used multi-contrast MRI to monitor the progression of grey and white matter injury in a mouse ICH model; (2) We used ADC and T2wt MRI to identify perihaematomal vasogenic oedema; (3) We investigated the association between perihaematomal oedema and glial response or iron deposition; (4) We analysed the correlation between DTI parameters (FA in CC, IC, and CP) and neurologic deficits; and (5) We compared Gd-enhanced T1wt MRI with Evans blue extravasation for assessment of BBB breakdown.

When measuring lesion volume, we obtained results similar to those of previous studies^16^,^22^. MRI and CV staining both showed that the lesion volume decreased from day 3 to day 7, with formation of a glial scar by day 28. These results show that MRI can be used to provide quantitative estimates of haemorrhagic lesion. We also found a correlation between MRI-determined lesion volume and NDS, indicating that MRI may be useful for evaluating the outcome of ICH and efficacy of treatments.

Brain oedema is another important marker of ICH severity and injury, and a reliable prognostic indicator. The increase in perihaematomal oedema volume was previously reported to increase the odds of poor outcome on discharge^23^. However, in our model, we did not detect cytotoxic oedema, which would have been evident as minimum T2 changes and a large decrease in ADC signals. Instead, we detected vasogenic oedema in the perihaematomal region, indicated by high T2 and high ADC signals, findings that support those of a prior human study^21^. Interestingly, we detected high T2 and low ADC signals in the lesion core, observations that differed from those in the human study^21^. The inconsistency may stem from differences in pathophysiology of the two ICH conditions. Clinically, ICH is caused by rupture of a tiny artery, which leads to formation of a haematoma or blood clot in the lesion core, with tissues pressed and distorted in the periphery^24^. In contrast, in the collagenase-induced ICH model, the lesion core is composed of blood, dead tissue, and cell debris because multiple vessels rupture over time. On days 3 and 7, the high T2 signal with low ADC signal at the lesion core indicated high water content but severely restricted water diffusion caused by the presence of blood and dead tissue. In the perihaematomal region, both the ADC value and the volume of regions with high-intense ADC values were higher on day 3 than on day 7, a finding supported by the measured striatal brain water content. We defined signal changes in other brain regions of the perihaematomal area based on the unique signatures on both T2wt and ADC signals. The low T2 signal and low ADC signal at lesion edge suggests fresh gel-like haematoma or tight tissue compressed by the perihaematomal oedema. The normal T2 signal and slightly high ADC signal at oedema edge is probably associated with the indirect effect of inflammation and BBB disruption. This information is important for understanding the formation of perihaematomal vasogenic oedema because it is challenging to measure directly with histologic methods.

Activated microglia and astrocytes and infiltrating macrophages are major inflammatory cells that contribute to secondary brain damage (e.g., brain oedema) after ICH^10^,^25^. Our histologic data showed that microglia/macrophage activation peaked in the perilesional region on day 3 and then decreased with time. The ADC signal intensities also increased in the same regions and at the same time point. Synchronous changes of microglia/macrophage activation and ADC signal intensities suggest that microglia/macrophage activation may lead to the formation of perihaematomal vasogenic oedema. Clearly, the cause and effect relation between the two should be investigated in the ICH model. Contrary to our expectations, changes in the number of astrocytes did not correlate with high ADC signals in the perihaematomal region, suggesting a limited contribution of astrocytes to the formation of perihaematomal vasogenic oedema. After microglia/macrophage activation, iron deposition in the ICH brain can cause oxidative brain injury^26^,^27^. In our study, iron deposition appeared on day 3 in the perilesional brain regions with high ADC signals but did not change synchronously with ADC signals, suggesting that iron deposition may not only contribute to the perihaematomal vasogenic oedema at the acute stage (day 3) but may also negatively affect brain repair at the subacute and chronic stage (day 7 and beyond).

Brain tissue loss is common in ICH models^15^,^17^,^19^,^28^,^29^,^30^,^31^. Most previous MRI studies have focused only on hemispheric brain tissue loss^19^,^28^,^29^,^30^,^31^ rather than atrophy in different brain regions. However, histology results show that brain atrophy occurs in several brain regions, including cortex^15^,^32^ and striatum^19^, and results in ventricular enlargement^28^. On T2wt MRI, we measured the volume of striatum, cortex, hippocampus, ventricle, and hemisphere to determine which brain regions change in volume because of long-term tissue loss after ICH. Our findings were similar to those of a previous study in rats^15^. Both MRI and histology showed that volumetric ratios of ipsilateral striatum and cortex to the contralateral hemisphere were significantly decreased at 28 days after ICH compared with those of sham controls. Hippocampus did not exhibit this change in ratio. Iron overload^33^,^34^,^35^, perihaematomal oedema, and inflammation-mediated cell death^19^,^28^,^29^ may contribute to brain atrophy because blocking these processes was shown to attenuate brain atrophy after ICH^33^,^34^.

Assessment of secondary white matter injury after ICH with DTI has gained attention recently because it has been reported that decreased FA of the pyramidal tract in the cerebral peduncles correlates with poor functional outcome^36^ and that higher FA measures of the ipsilateral posterior limb of the CC predict better motor recovery in stroke-affected upper limb^9^. DTI/MRI has been investigated as a promising technique to evaluate functional outcome and compare treatment effects in patients with ICH^37^. Only a few preclinical ICH studies have used FA to estimate white matter injury and repair^18^,^19^,^38^. The first study showed decreased FA value at day 3 together with histologically determined neuronal degeneration, which continued to 120 days after collagenase-induced ICH in rats^18^. The second study, which used the blood-injection ICH model in rats, also showed a decrease in FA in the tissue near the haemorrhagic region on days 1 and 3 but did not present longer-term data^38^. We were the first group to show decreases in FA values of the ipsilateral CC on day 28 in a mouse collagenase-induced ICH model^19^. Our results here show similar changes of FA values in CC and IC, both of which are common locations measured in ICH animal studies^4^,^15^. We obtained similar findings, perhaps because pyramidal tracts comprise fibres that travel though the IC. The novel data with *ex vivo* DTI in our study showed marked fibre loss in the ipsilateral CC at day 28. MacLellan *et al*.^15^ reported that CC volume was decreased at 6 weeks after ICH compared with that of contralateral CC. Our results further show a negative correlation between FA value in the ipsilateral IC and NDS, though it disappeared after we adjusted for total lesion volume on T2wt MRI. The FA value in CP decreased at day 3 and then remained the same level until day 28 after ICH. A recent clinical study showed similar results^39^. We did not find a significant correlation between FA value in the ipsilateral CP and NDS. Interestingly, two clinical studies^36^,^39^ showed that the FA value in the ipsilateral CP early after ICH might predict motor outcome at the chronic stage. Our results differ from theirs, most likely because the CP in mouse brain is so small.

Disruption of the BBB is associated with brain oedema^40^ and white matter injury^41^,^42^, which contribute to secondary injury in ICH^14^,^15^,^43^,^44^. MacLellan *et al*.^15^ reported a time-dependent (12 h, 2 and 4 days) increase in BBB permeability of rats subjected to the collagenase model, but not the blood-injection model. We used the collagenase-induced ICH model and evaluated BBB permeability for a longer time course. T1-Gd MRI signal intensity increased beginning at day 1 and peaked on day 3 before decreasing at day 7 and almost disappearing at day 11. These findings, which were validated by Evans blue staining, show that BBB breakdown has begun on day 1, peaks on day 3, begins to recover on day 7, and is fully recovered by day 11. Increased BBB disruption may result in perihaematomal vasogenic oedema, which also peaks at day 3. Liu *et al*.^40^ reported the same trend of Evans blue staining in a thrombin-induced ICH model, showing that BBB is severely damaged by 1 day after thrombin injection but completely recovered by 14 days. The authors explained that Src kinases mediate both acute BBB injury and chronic BBB repair after thrombin-induced brain injury^40^.

Our current study shows that multimodality MRI can be used to track the dynamic progression of post-ICH injury, including haemorrhagic lesion, vasogenic brain oedema, white matter injury, and BBB breakdown, and that the results correspond with histologic findings and correlate with neurologic function. We conclude that these MRI scanning methods can be used to evaluate therapeutic interventions in preclinical ICH models and eventually humans for precision-medicine-based clinical care. Additionally, our findings indicate that increased vasogenic oedema in the perihaematomal region might be associated with microglial activation, iron toxicity, and BBB disruption.

## Methods and Materials

### Animals

All animal studies were conducted in accordance with National Institutes of Health guidelines and were approved by the Johns Hopkins University Animal Care and Use Committee. C57BL/6 male mice (12 months old, 32–36 g) were obtained from Charles River Laboratories (Frederick, MD) and maintained in the Johns Hopkins animal facility. All efforts were made to minimize the number of animals used and their suffering. Animal experiments were reported in accordance with the ARRIVE guidelines.

### ICH mouse model

ICH was induced by injecting collagenase into the left striatum of mice according to a previously described method^19^,^45^. Briefly, mice were placed in a stereotaxic frame under anaesthesia with isoflurane. A burr hole was drilled into the skull, and type VII-S collagenase (0.075 U in 0.5 μl saline; Sigma, St. Louis, MO) was infused into the left striatum with a microinfusion pump at a constant rate of 0.1 μl/min. The needle tip was placed for haematoma induction to invade the middle of the striatum without damaging the motor cortex: 0.4 mm anterior and 2.0 mm lateral from the bregma, 3.0 mm below the skull. Sham-operated mice were infused with an equal volume of saline. Body temperature was maintained at 37 ± 0.5 °C during surgery. After surgery, mice were returned to their home cage. Vital conditions of the mice were monitored every day after ICH surgery.

### Histology

Mice were anesthetized with isoflurane at day 3, 7, or 28 after ICH and perfused transcardially with phosphate-buffered saline (PBS) followed by 4% paraformaldehyde. Brains were isolated and fixed in 4% paraformaldehyde overnight and then soaked in 30% sucrose for 72 h at 4 °C. Coronal sections (25 μm) were taken at 150 μm intervals from +1.2 to −2.4 mm from the bregma. Sections were mounted onto slides and stained with CV for measurement of lesion and brain region volumes, and with LFB for measurement of myelin integrity. Perls’ staining was used to detect iron deposition. Additional adjacent sections were used for immunochemical staining as described below. Changes in brain regions were defined as (brain region volume/contralateral hemispheric volume) × 100%, as described previously^15^,^28^.

### Immunohistochemistry

Before primary antibody was applied, all sections were blocked in PBS containing 3% normal goat serum and 0.2% Triton X-100 for 60 min at room temperature. Both primary and secondary antibody were diluted in PBS containing 3% normal goat serum. Based on our established protocol^46^, to label astrocytes and microglia/macrophages, we incubated free-floating cryosections for 12 h at 4 °C in a dilution of anti-mouse glial fibrillary acidic protein (GFAP) antibody (1:500; Sigma) and anti-rabbit Iba-1 antibody (1:500; Wako Chemicals, Richmond, VA). GFAP-positive cells were visualized with a green fluorochrome-tagged secondary antibody (Cy2, 1:1000; Invitrogen, Waltham, MA), and Iba-1–positive cells were visualized with a red fluorochrome-tagged secondary antibody (Alexa 594, 1:1000; Invitrogen) after incubation at room temperature for 1 h.

### Quantification of Perls’ staining and immunofluorescence

Stained sections were examined with a Nikon Eclipse 90i fluorescence microscope (Nikon, Tokyo, Japan). We examined four randomly selected fields at 200× magnification in three sections with similar haematoma sizes in each animal. Quantifications from 12 locations were averaged and expressed as positive cells (Iba1-, GFAP-) or positive areas (Perls’ staining) with Image J software (NIH, Image J 1.48 v) as previously described^19^,^47^.

### Transmission electron microscopy

At 3 days of recovery, sham and ICH mice were perfused with 2% paraformaldehyde and 2% glutaraldehyde in 0.1 M sodium cacodylate buffer, and then post-fixed in 2% osmium tetroxide with 1.6% potassium ferrocyanide in 0.1 M sodium cacodylate. Samples (at the margin of the haematoma or corresponding location) were then cut and stained *en bloc* with 2% uranyl acetate, dehydrated in ethanol, and embedded in eponate. Then the sections (70–90 nm) were placed on copper slot grids and stained with 2% uranyl acetate and lead citrate. Transmission electron microscopy images were captured with a Hitachi 7600 in the microscope core of Johns Hopkins University.

### MRI

We performed *in vivo* MRI experiments on a horizontal 11.7 Tesla MR scanner (Bruker Biospin, Billerica, MA, USA) equipped with triple-axis gradient (maximum gradient strength = 74 Gauss/cm) using a volume excitation coil and a 4-channel phased array mouse head receive-only coil. Animals were anesthetized with 1–1.5% isoflurane in a mix of oxygen and air at a 1:3 ratio and placed in an animal holder (Bruker Biospin). Respiration was monitored with a pressure sensor (SAII, Stony Brook, NY, USA) and maintained at 50–60 breaths per minute by adjusting the concentration of isoflurane. Multi-slice T2wt images were collected by using a rapid acquisition with relaxation enhancement (RARE) sequence with the following parameters: echo time/repetition time = 60/3800 ms, RARE-factor = 8, four signal averages, field of view = 15 mm × 15 mm, 28 slices with 0.5 mm slice thickness, in-plane resolution of 0.08 mm × 0.08 mm, and an imaging time of 12 min. Multi-slice T1-weighted (T1wt) images were collected by using a spin echo sequence with the following parameters: echo time/repetition time = 9/250 ms, four signal averages, field of view = 15 mm × 15 mm, 10 slices with 0.5 mm slice thickness, in-plane resolution of 0.12 mm × 0.12 mm, and an imaging time of 10 min. Pre-contrast T1wt images were acquired before the injection of Gd- diethylenetriaminepentacetate (DTPA) (Magnevist, Bayer, Whippany, NJ). Post-contrast T1wt images were acquired 10 min after intraperitoneal injection of Gd-DTPA (0.2 ml/kg body weight) with normal saline. Multi-slice *in vivo* DTI was performed by using a four-segment diffusion-weighted echo-planar imaging sequence with the following parameters: echo time/repetition time = 24/14000 ms; one signal average; 30 diffusion directions; b = 1500 s/mm^2^; an in-plane resolution of 0.12 mm × 0.12 mm with a partial Fourier factor of 1.4 in the phase-encoding direction, the same field of view, slice thickness, and number of slices as the T2-weighted images. With respiratory gating, the total imaging time was approximately 30 min. All animals recovered in 5 min after imaging. We reconstructed the diffusion tensor at each pixel along with ADC and FA using the log-linear fitting method implemented in DTIStudio (http://www.mristudio.org).

In mice that had undergone collagenase-induced ICH, we manually defined the haematoma volume on days 3 and 7, residual lesion volume on day 28, and volume of striatum, hemisphere, ventricle, hippocampus, and cortex at all three time points in the T2wt images using ROIEditor (http://www.mristudio.org)^19^. White matter injury on day 28 post-ICH was analysed from DTI. We manually defined the ipsilateral CC from the midline to the lateral edge of the lateral ventricles in the FA images using ROIEditor and obtained the average FA value in the region for each subject.

### Quantification of Evans blue leakage

A 2% solution of Evans blue in normal saline (4 ml/kg of bodyweight) was injected through the tail vein as described previously^48^. The stain was allowed to circulate for 2 h. Then the mice were perfused transcardially with PBS, and the brain tissue was removed and divided into right and left hemispheres. Each part of the brain was homogenized in 1 ml of PBS and then sonicated and centrifuged (30 min, 10,000 rcf, 4 °C). Evans blue stain was measured by spectrophotometry at 620 nm and quantified according to a standard curve. The results are presented as ratio of left to right hemisphere.

### NDS

On days 3, 7, and 28 after surgery, an investigator blinded to group assignment evaluated the mice by an NDS system that included six domains of body symmetry, gait, climbing, circling behaviour, front limb symmetry, and compulsory circling. Each test was graded from 0 to 4, providing a total score of 0 to 24, with higher scores indicative of greater neurologic injury^49^,^50^.

### Statistical analysis

All data are reported as mean ± SD. Data were analysed with Student’s *t* test or one-way ANOVA. Linear regression was used for correlational analysis between MRI and histological estimates of haemorrhagic lesion, brain atrophy, and oedema. A *P*-value < 0.05 was considered statistically significant. All statistical analyses were performed with IBM SPSS Statistics 21 (IBM, Armonk, NY).

## Additional Information

**How to cite this article**: Yang, J. *et al*. Multimodality MRI assessment of grey and white matter injury and blood-brain barrier disruption after intracerebral haemorrhage in mice. *Sci. Rep.* **7**, 40358; doi: 10.1038/srep40358 (2017).

**Publisher's note:** Springer Nature remains neutral with regard to jurisdictional claims in published maps and institutional affiliations.

## Supplementary Material

Supplementary Figures
