# Supplementary material for: Multimodality MRI assessment of grey and white matter injury and blood-brain barrier disruption after intracerebral haemorrhage in mice
Source: Sci Rep. 2017 Jan 13;7:40358. doi: 10.1038/srep40358 (PMC5234017; doi:10.1038/srep40358)
Supplement: Supplementary Figures [file srep40358-s1.pdf]

Scientific Reports (Regular Article) -**Supplementary materials**

Multimodality MRI assessment of grey and white matter injury and blood-brain barrier disruption after intracerebral haemorrhage in mice

Jie Yang[1,2], Qian Li[2], Zhongyu Wang[2], Cunfang Qi[2], Xiaoning Han[2], Xi Lan[2], Jieru Wan[2], Wenzhu Wang[2], Xiaochun Zhao[2], Zhipeng Hou[3], Cong Gao[1], J. Ricardo Carhuapoma[2,4], Susumu Mori[3], Jiangyang Zhang[5*], Jian Wang[2*]

[1]Key Laboratory of Neurogenetics and Channelopathies of Guangdong Province and The Ministry of Education of China, Institute of Neuroscience; Department of Neurology, the Second Affiliated Hospital of Guangzhou Medical University, Guangzhou, China.

[2]Department of Anesthesiology/Critical Care Medicine, The Johns Hopkins University, School of Medicine, Baltimore, MD 21205, USA.

[3]Department of Radiology, The Johns Hopkins University School of Medicine, Baltimore, MD 21205, USA

[4]Department of Neurology, The Johns Hopkins University School of Medicine, Baltimore, MD 21205, USA

[5]Bernard and Irene Schwartz Center for Biomedical Imaging, Department of Radiology, New York University School of Medicine, New York, NY 10016, USA.

Supplementary Figure 1

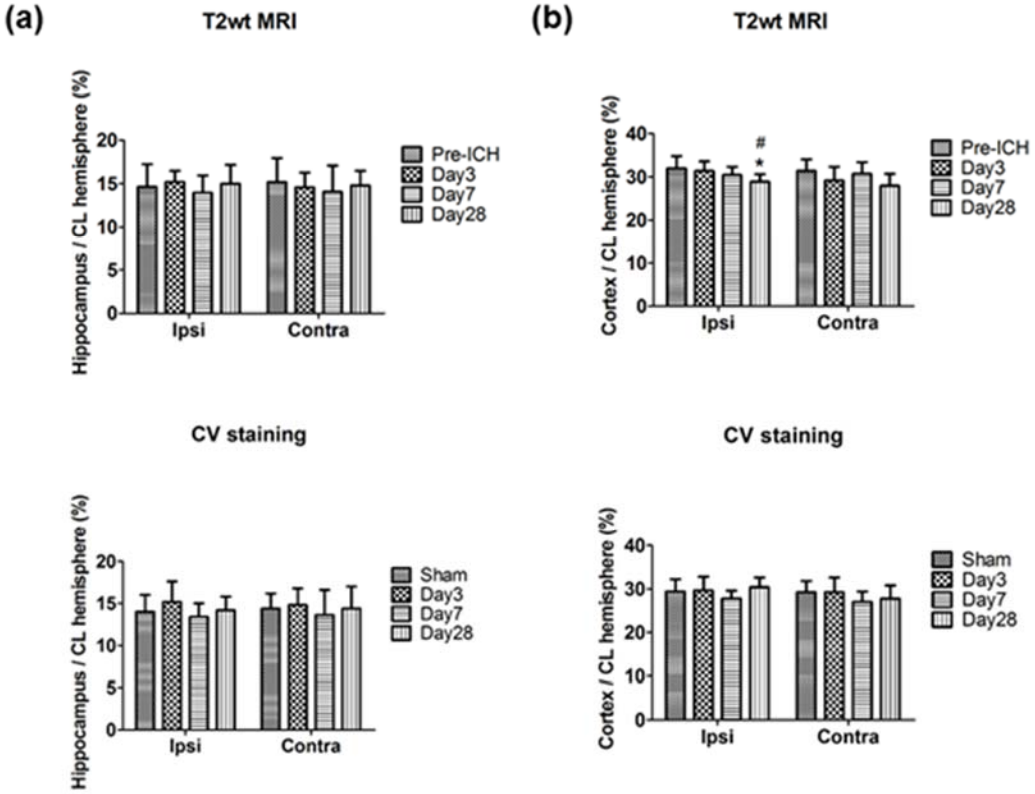

Supplementary Figure 1. Changes in brain region volume over time in hippocampus and cortex. The ratios of bilateral hippocampus and cortex to the contralateral hemisphere did not change significantly over time after ICH as assessed by T2 MRI (**a, b** top panels) or cresyl violet (CV) staining of brain sections (**a, b** bottom panels). T2wt MRI: n=5 for pre-ICH and n=8 at other time points; CV staining: n=6 at each time point (*P<0.05 vs. pre-ICH; #P<0.05 vs. day 3, one-way ANOVA). CL = contralateral.

Supplementary Figure 2

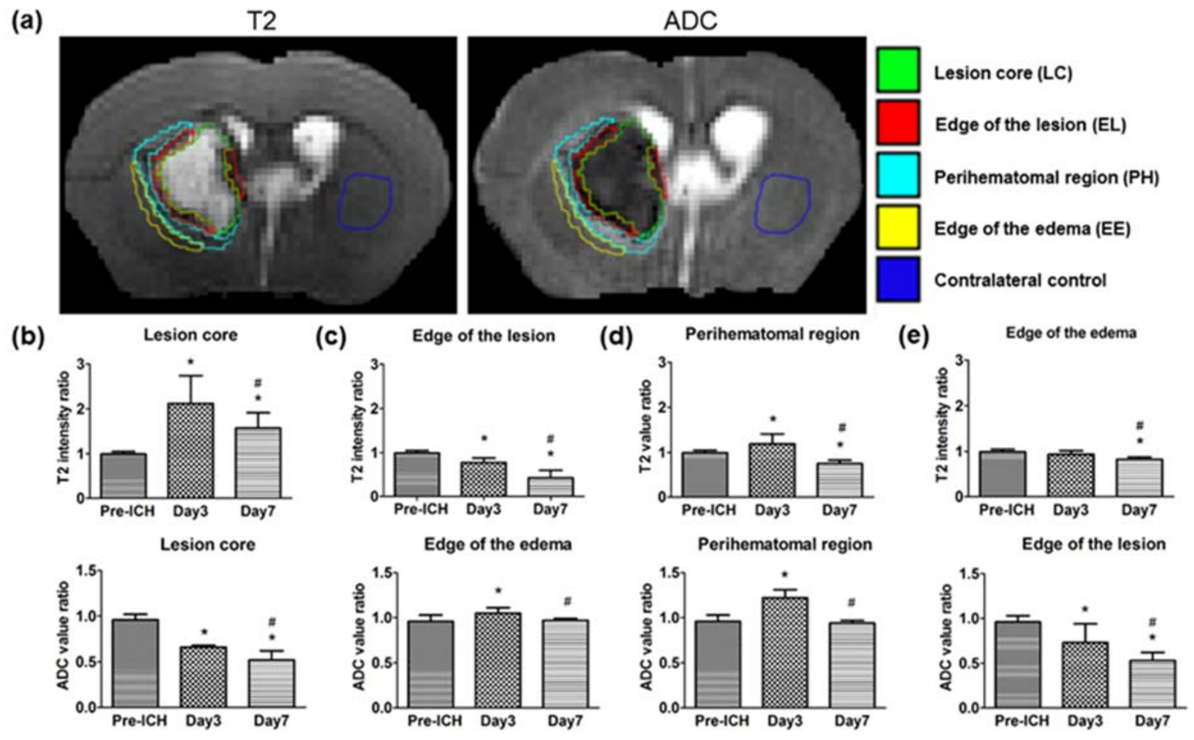

Supplementary Figure 2. T2 and ADC signal changes in both lesion and perilesional area. (**a**) Based on unique signatures in MRI signals, the combination of the T2 and ADC signal intensity identified four regions with different pathologic states and was able to monitor the lesion progression longitudinally. These regions include lesion core (LC, green outline), edge of the lesion (EL, red), perihaematomal region (PH, blue), and edge of the oedema (EE, yellow). The region with purple outline is the contralateral control region. The MR signatures are tabulated below.

	LC	EL	PH	EE
T2	Increase	Decrease	Increase	No change
ADC	Decrease	Decrease	Increase	Slight increase

LC indicates impeded water mobility caused by the high viscosity. EL suggests tight tissue compressed by the perihaematomal oedema. PH shows the vasogenic oedema associated with microglial activation, iron deposition, and BBB disruption. EE is possibly associated with the indirect effect of neuroinflammation and BBB disruption. We used the ratio of values in the first

four areas to that in the contralateral brain tissue on T2 or ADC images to show the signal changes. (**b**) In LC, theT2 ratio increased at day 3 and decreased at day 7 but remained higher than that at pre-ICH at both time points. The ADC ratio decreased from day 3 to day 7 compared to that at pre-ICH. (**c**) In EL, the ratio of both T2 and ADC value decreased significantly from day 3 to day 7 compared to that at pre-ICH. (**d**) In PH, the T2 ratio was elevated on day 3, but decreased on day 7, compared with that at baseline. The ADC ratio was increased on day 3, but not on day 7, compared with that at pre-ICH, indicating that perihaematomal vasogenic oedema increased initially but was gone by day 7. (**e**) In EE, the T2 ratio did not change at day 3 but decreased at day 7 compared to that at pre-ICH. The ADC ratio increased significantly at day 3 but decreased at day 7 compared to that at pre-ICH. n=5 for pre-ICH and n=8 at other time points (*$P<0.05$ vs. pre-ICH; #$P<0.05$ vs. day 3, one-way ANOVA).

Supplementary Figure 3

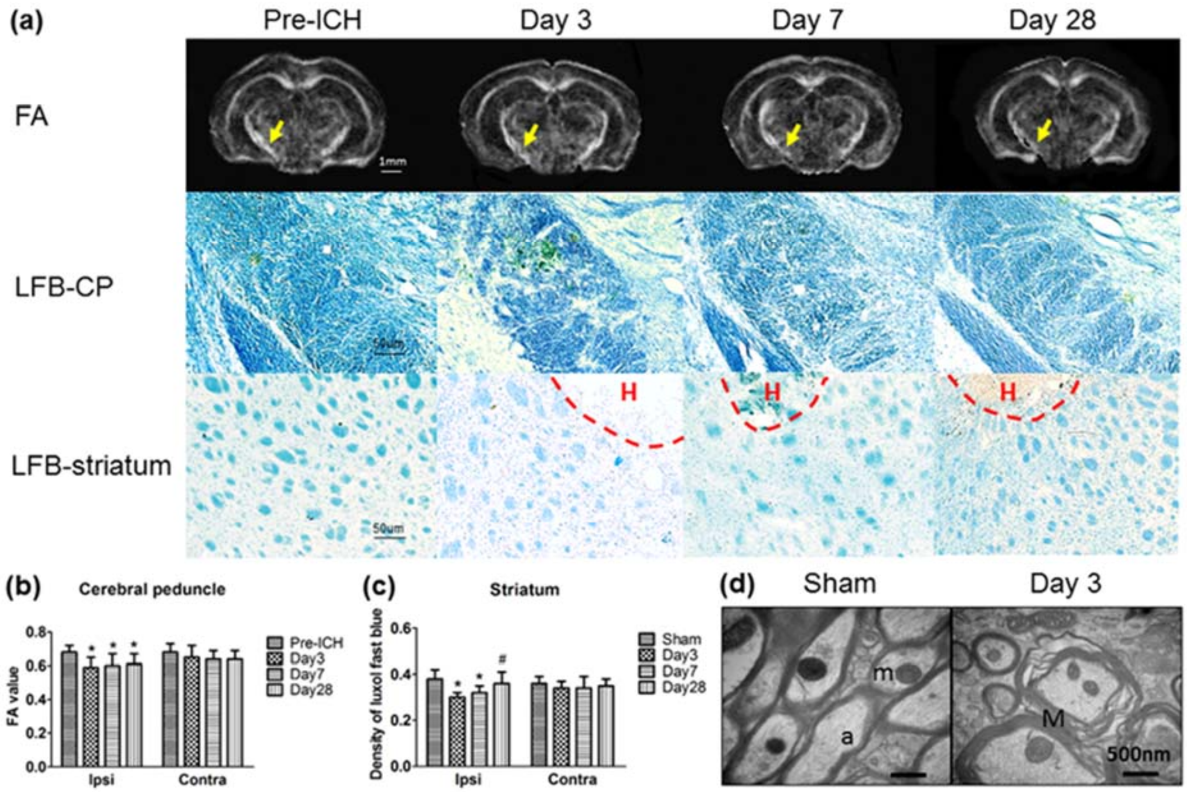

Supplementary Figure 3. White matter injury in cerebral peduncle (CP) and striatum. (**a**) The DTI image shows the impaired ipsilateral CP (yellow arrow) at days 3, 7, and 28 compared to the intact CP at pre-ICH (top). Fast blue staining shows injured myelin in the ipsilateral CP (middle) and striatum (bottom) at those time points after ICH. (**b**) Quantitative analysis showed decreased fractional anisotropy (FA) value in CP from day 3 to day 28 post-ICH compared to that in the sham group. (**c**) The intensity of fast blue staining was decreased at days 3 and 7, but it increased back to the level of the sham group at day 28. (**d**) Transmission electron microscopy shows marked pathologic changes in myelin structure characterized by myelin breakdown or disruption at day 3 post-ICH and intact myelin in the sham mouse brain. M: myelin; m: mitochondria; a: axon. DTI: n=5 for pre-ICH and n=8 at other time points; Luxol fast blue staining: n=1 for CP and n=6 for striatum at each time point; transmission electron microscopy: n=3 for each (*P<0.05 vs. pre-ICH for DTI and vs. sham for fast blue staining; #P<0.05 vs. day 3, one-way ANOVA).
